# Genomic analysis of porcine circovirus type 2 from southern China

**DOI:** 10.1002/vms3.288

**Published:** 2020-06-08

**Authors:** Qizhuang Lv, Tao Wang, Jiahua Deng, Yan Chen, Qiu Yan, Daobo Wang, Yulin Zhu

**Affiliations:** ^1^ College of Biology & Pharmacy Yulin Normal University Yulin PR China; ^2^ Guangxi Key Laboratory of Agricultural Resources Chemistry and Biotechnology Yulin PR China; ^3^ College of Veterinary Medicine Northwest A&F University Yangling PR China

**Keywords:** capsid protein, genetic variation, phylogenetic analysis, porcine circovirus type 2, recombination

## Abstract

**Background:**

Porcine circovirus type 2 (PCV2) is recognized as virulent porcine pathogen and has been linked to porcine circovirus diseases (PCVD). However, there remain many unknowns regarding the spread and epidemic growth of PCV2.

**Methods:**

To assess the genetic diversity of PCV2 in the southern China, a total of 92 sequences of PCV2 strains from this region were retrieved from GenBank and were subjected to amino acid variation and phylogenetic analyses together with 28 representative sequences, based on the sequence of the ORF2 gene, from different swine‐producing countries.

**Results:**

All 92 PCV2 strains shared between 93.7% and 100% sequence similarity and could be divided into four genotypes (PCV2a, PCV2b, PCV2d and PCV2h), of which PCV2d had surpassed PCV2b and became the most prevalent PCV2 genotype in this region. Alignment of the deduced amino acid sequences of the capsid protein revealed that the obtained PCV2 strains possess two major heterogenic regions/hypervariable regions (positions 52–68 and 185–191), which were within or close to the epitopic regions in the capsid (Cap) protein. Meanwhile, the 92 PCV2 sequences also show evidence of at least five unique recombination events.

**Conclusion:**

The data in this study indicate that the PCV2 strains in the southern China are undergoing constant genetic variation and that the predominant strain and its antigenic epitopes in this area have been gradually changing in recent years.

## Graphical abstract

PCV2 strains from the Beibu Gulf of China could be divided into four genotypes (PCV2a, PCV2b, PCV2d and PCV2h), of which PCV2d had surpassed PCV2b and became the most prevalent PCV2 genotype in this area. In addition, the Beibu Gulf PCV2 strains possess two major heterogenic regions/hypervariable regions (positions 52–68 and 185–191), which were within or close to the epitopic regions in the capsid (Cap) protein. Meanwhile, these PCV2 sequences also show evidence of at least five unique recombination events.

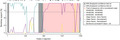

## 1 INTRODUCTION

*Porcine circovirus type 2* (PCV2), a member of the genus *Circovirus* of the *Circoviridae* family, is the smallest virus known to infect mammals. Increasing evidences indicate that PCV2 is the primary causative agent of PCV2‐systemic disease (PCV2‐SD) and PCV2‐subclinical infection (PCV2‐SI), and is associated with other pathological conditions designated by porcine circovirus diseases (PCVD), which cause a relevant economic impact in the global swine industry (Segalés, 2015). As a non‐enveloped, single‐stranded DNA virus, the PCV2 virion is icosahedral and 17 nm in diameter (Lv, Guo, & Zhang, 2014). The PCV2 has an ambisense, closed, circular genome with a size of 1,766–1,768 and 1,777 nucleotides that is computationally predicted to possess 11 overlapping open reading frames (ORFs) (Nguyen et al., 2012). To date, six ORFs have been characterized in detail: ORF1 codes for two replication‐associated proteins (Rep and Rep'), ORF2 codes for the capsid protein (Cap)involved in the host immune response, ORF3 codes for the apoptotic protein, ORF4 codes for the anti‐apoptotic protein (Lv, Guo, Zhang, & Zhang, 2016), ORF5 codes for a novel potentially endoplasmic reticulum stress‐inductive protein (Lv, Guo, Xu, Wang, & Zhang, 2015) and ORF6 codes for a newly discovered protein that may be involved in caspases regulation and the expression of multiple cytokines in PCV2‐infected cells (Li, Wang, et al., 2018; Li, He, et al., 2018). Particularly, ORF2 is a common target gene used for epidemiological and phylogenetic analyses on PCV2 strains as the analysis has been shown to be representative of full genome analysis. Previous studies have substantiated that the Cap protein encoded by ORF2 possesses three specific antigenic sites (aa 69–83, aa 117–131 and aa 169–183) and three spatial overlapping antigenic epitopes (aa 47–63, aa 165–200 and aa 230–233) (Lv et al., 2014).

PCV2 can be divided into eight genotypes (PCV2a to PCV2h), which were compiled based on the updated phylogeny‐grounded genotype definition for PCV2 strains that have been described by Franzo and Segalés (2018). PCV2a and PCV2b are the most common strains. In approximately 2003, there was a global‐scale shift in PCV2 from genotype PCV2a to PCV2b, which is highly prevalent in many countries. PCV2c is a genotype that was first identified in Denmark (Dupont, Nielsen, Baekbo, & Larsen, 2008), while PCV2d and PCV2e are two later discovered genotypes in China and other countries (Guo, Lu, Wei, Huang, & Liu, 2010; Wang et al., 2009). PCV2f is a novel genotype that was first identified by Bao et al. (2018), while PCV2g and PCV2h are two more genotypes that are recently proposed by Franzo and Segalés (2018). Additionally, PCV2 might be divided into group 1 (PCV2b) and group 2 (PCV2a) with eight clusters (1A–1C and 2A–2E) in another PCV2 genotype definition (Olvera, Cortey, & Segalés, 2007). The link between PCV2 group and disease status has been investigated many times but the results are not clear‐cut. Of these results, it is generally accepted that PCV2a, PCV2b and PCV2d are able to experimentally reproduce PCV2‐SD under appropriate circumstances, such as co‐infection with other swine pathogens or immunostimulation by vaccines or adjuvants (Gillespie, Opriessnig, Meng, Pelzer, & Buechner‐Maxwell, 2009; Opriessnig et al., 2014; Segalés, 2015).

In China, PCV2 infection was first recognized in 1996, and was then subsequently identified in most pig farms in different regions (Bao et al., 2018). Currently, several studies has revealed that Chinese strains share a high‐nucleotide sequence identity and mainly belong to genotypes PCV2b andPCV2d, with PCV2d becoming more dominant (Liu et al., 2018). There are known recombinant events of PCV2 within the ORF2 gene that are considered to be type‐specific and closely related to the pathogenesis (Cai et al., 2012; Wang et al., 2009). However, there remain many unknowns regarding the spread and epidemic growth of PCV2. In the present study, we obtained the complete genomes of 92 PCV2 strains from the Guangxi Beibu Gulf economic zone in China from GenBank and subjected them to amino acid variation and phylogenetic analyses, along with 28 PCV2 strains from different geographic regions throughout the world, based on the sequence of the ORF2. This information may provide valuable insights in to the genetic variation and phylogenetic characteristics of PCV2 populations circulating in this area and shed new light on the choice of vaccines that are ultimately used.

## 2 MATERIALS AND METHODS

### 2.1 Collection of PCV2 genome sequence

**FIGURE 1:**
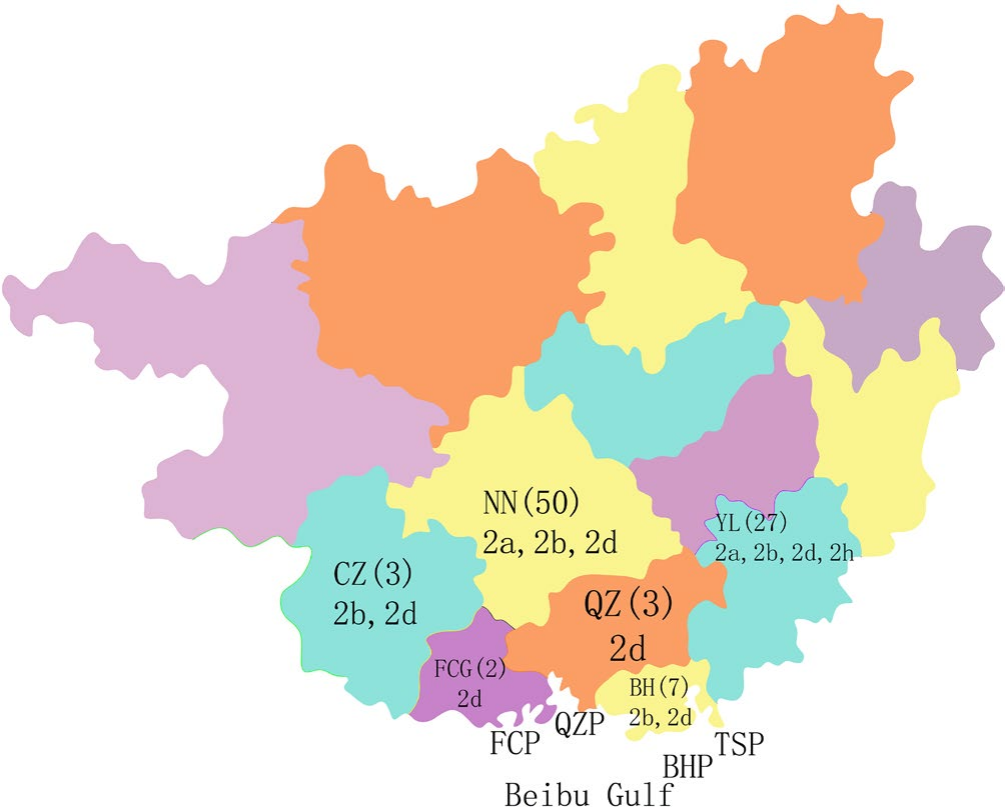
Geographical distribution of archived PCV2 strains. The abbreviations represent names of regions, the numbers represent the number of PCV2 strains and the genotypes detected in each region are also indicated under the numbers

A total of 92 PCV2 full‐genome sequences were collected from different regions of the Guangxi Beibu Gulf economic zone in China from 2006 to 2016. Meanwhile, an additional 28 PCV2 strains derived from other countries or regions were retrieved from GenBank. Specifically, a significant amount of PCV2 strains were isolated from sick pigs with various clinical syndromes, such as postweaning multisystemic wasting syndrome (PMWS), respiratory signs, wasting, death etc, although the small number of strains are unpublished or their clinical status of the host have not been specifed. The information on geographical origin, year of strain isolation and genotype/subgroup are summarized in Tables 1 and 2. The geographical distribution of the 92 samples is shown in Figure 1. To facilitate the analysis, all of these obtained sequences were linearized at the same point and aligned with the Clustal W component of the MegAlign program (DNASTAR, version 7.10) as described previously (Mu et al., 2012).

**TABLE 1:** PCV2 strains isolated from the southern China used in this study

Isolate's name	Year of isolation	Genome size (nt)	Genotype	Accession number	References
Huanan‐10	2006	1,767	PCV2b	EF493839	unpublished
Huanan‐9	2006	1,767	PCV2b	EF493838	unpublished
GX0601	2006	1,768	PCV2a	EF524532	Wang et al., 2009
GX0602	2006	1,768	PCV2a	EF524533	Wang et al., 2009
GXNN0603	2006	1,767	PCV2b	MH465418	Yao et al., 2019
GXYL0601	2006	1,767	PCV2b	MH465433	Yao et al., 2019
GXWM	2007	1,767	PCV2d	EF675241	Huang et al., 2006
GXLC	2007	1,767	PCV2b	EF675240	Yin et al., 2007
GXHP	2007	1,767	PCV2b	EF675239	Huang et al., 2006
GXHK	2007	1,767	PCV2b	EF675238	Huang et al., 2006
GXGW	2007	1,767	PCV2b	EF675237	Huang et al., 2006
GXNN0804	2008	1,767	PCV2d	MH465458	Yao et al., 2019
GXNN0806	2008	1,767	PCV2b	MH465420	Yao et al., 2019
GXNN0803	2008	1,767	PCV2b	MH465419	Yao et al., 2019
GXBH0801	2008	1,767	PCV2b	MH465398	unpublished
GXCZ0805	2008	1,767	PCV2b	MH465402	Yao et al., 2019
GXNN0904	2009	1,767	PCV2d	MH465460	Yao et al., 2019
GXNN0901b	2009	1,767	PCV2d	MH465459	Yao et al., 2019
GXNN0902	2009	1,767	PCV2b	MH465422	Yao et al., 2019
GXNN0901a	2009	1,767	PCV2b	MH465421	Yao et al., 2019
GXBH1008	2010	1,767	PCV2b	MH465399	Yao et al., 2019
GXFC11	2011	1,767	PCV2d	KJ680370	unpublished
BL12	2012	1,767	PCV2d	KJ680369	unpublished
BLFC12	2012	1,767	PCV2d	KJ680368	unpublished
GXYQ12	2012	1,767	PCV2d	KJ680367	unpublished
GXNN1209b	2012	1,767	PCV2b	MH465424	Yao et al., 2019
GXNN1209a	2012	1,767	PCV2b	MH465423	Yao et al., 2019
GXWM121203	2012	1,767	PCV2d	MH756618	unpublished
GXSL121231	2012	1,767	PCV2d	MH756617	unpublished
GXYL1208	2012	1,767	PCV2h	MH465473	Yao et al., 2019
GXNN1304a	2013	1,767	PCV2d	MH465461	Yao et al., 2019
GXNN1312	2013	1,767	PCV2b	MH465426	Yao et al., 2019
GXNN1304b	2013	1,767	PCV2b	MH465425	Yao et al., 2019
GXNN130121	2013	1,767	PCV2b	MH756615	unpublished
GXLA130701	2013	1,767	PCV2d	MH756613	unpublished
GXBB130624	2013	1,767	PCV2d	MH756608	unpublished
GXBL130228	2013	1,767	PCV2d	MH756610	unpublished
GXBL130313	2013	1,767	PCV2d	MH756611	unpublished
GXYL1307c	2013	1,767	PCV2d	MH465475	Yao et al., 2019
GXYL1307a	2013	1,767	PCV2d	MH465474	Yao et al., 2019
GXYL1310	2013	1,767	PCV2b	MH465437	Yao et al., 2019
GXYL1307d	2013	1,767	PCV2b	MH465436	Yao et al., 2019
GXYL1305	2013	1,767	PCV2b	MH465435	Yao et al., 2019
GXYL1304	2013	1,767	PCV2b	MH465434	Yao et al., 2019
GXYL1307b	2013	1,768	PCV2a	MH465490	Yao et al., 2019
BH5	2014	1,767	PCV2d	KM245558	Zhang et al., 2017
NN2	2014	1,767	PCV2b	KJ956692	Zhang et al., 2017
HX02	2014	1,767	PCV2b	KJ956691	Zhang et al., 2017
HX1	2014	1,767	PCV2b	KJ956690	Zhang et al., 2017
BH6	2014	1,767	PCV2b	KJ956689	Zhang et al., 2017
GXNN1410c	2014	1,767	PCV2d	MH465464	Yao et al., 2019
GXNN1410a	2014	1,767	PCV2d	MH465463	Yao et al., 2019
GXNN1409a	2014	1,767	PCV2d	MH465462	Yao et al., 2019
GXNN1406	2014	1,767	PCV2b	MH465427	Yao et al., 2019
BH7	2014	1,767	PCV2d	KY305198	unpublished
GXNN141225	2014	1,767	PCV2d	MH756616	unpublished
GXLA140815	2014	1,767	PCV2d	MH756614	unpublished
GXYL1410	2014	1,767	PCV2d	MH465480	Yao et al., 2019
GXYL1405	2014	1,767	PCV2d	MH465479	Yao et al., 2019
GXYL1403b	2014	1,767	PCV2d	MH465478	Yao et al., 2019
GXYL1403a	2014	1,767	PCV2d	MH465477	Yao et al., 2019
GX140420	2014	1,767	PCV2d	MH756607	unpublished
GXYL1401	2014	1,767	PCV2d	MH465476	Yao et al., 2019
GXYL1409	2014	1,767	PCV2b	MH465438	Yao et al., 2019
GXCZ1410	2014	1,767	PCV2b	MH465403	Yao et al., 2019
GXNN1410b	2014	1,768	PCV2a	MH465488	Yao et al., 2019
GXNN1409b	2014	1,768	PCV2a	MH465487	Yao et al., 2019
GXYL1408	2014	1,767	PCV2a	MH465491	Yao et al., 2019
GXNN1504	2015	1,767	PCV2d	MH465467	Yao et al., 2019
GXNN1503	2015	1,767	PCV2d	MH465466	Yao et al., 2019
GXNN1501	2015	1,767	PCV2d	MH465465	Yao et al., 2019
GXNN1511	2015	1,767	PCV2b	MH465428	Yao et al., 2019
GXNN5	2015	1,767	PCV2b	KY305202	unpublished
GXNN3	2015	1,767	PCV2b	KY305201	unpublished
GXQZ2	2015	1,767	PCV2d	KY305200	unpublished
GXQZ1	2015	1,767	PCV2d	KY305199	unpublished
GXYL1512	2015	1,767	PCV2d	MH465481	Yao et al., 2019
GXBB1501211	2015	1,767	PCV2d	MH756609	unpublished
GXFC1501	2015	1,767	PCV2d	MH465443	Yao et al., 2019
GXCZ1510b	2015	1,767	PCV2d	MH465442	Yao et al., 2019
GXNN1612b	2016	1,767	PCV2d	MH465471	Yao et al., 2019
GXNN1612a	2016	1,767	PCV2d	MH465470	Yao et al., 2019
GXNN1603a	2016	1,767	PCV2d	MH465469	Yao et al., 2019
GXNN1602	2016	1,767	PCV2d	MH465468	Yao et al., 2019
GXNN1612c	2016	1,767	PCV2b	MH465431	Yao et al., 2019
GXNN1604b	2016	1,767	PCV2b	MH465430	Yao et al., 2019
GXNN1603b	2016	1,767	PCV2b	MH465429	Yao et al., 2019
GXNN2	2016	1,767	PCV2d	KY305204	unpublished
GXNN1	2016	1,767	PCV2d	KY305203	unpublished
GXYL1607	2016	1,767	PCV2d	MH465482	Yao et al., 2019
GXQZ1601	2016	1,767	PCV2d	MH465472	Yao et al., 2019
GXNN1604a	2016	1,768	PCV2a	MH465489	Yao et al., 2019

**TABLE 2:** Representative PCV2 strains used in the genetic variation and phylogenetic analyses

Isolate's name	Year of isolation	Geographic origin	Genome size (nt)	Group/Cluster	Accession number	References
NAVET_vietnam3	2004	Vietnam	1,767	PCV2h	JX506730	Franzo & Segalés, 2018
FJ	2004	Fujian‐China	1,768	PCV2a	AY556474	Nguyen et al., 2012
BJW	2004	Beijing‐China	1,767	PCV2b	AY847748	Olvera et al., 2007
SCNB/CHA/05	2005	Sichuan‐China	1,767	PCV2g	FJ998185	Franzo & Segalés, 2018
USA/MN‐088/2006	2006	USA	717 ORF2 gene	PCV2e	KT867799	Franzo & Segalés, 2018
JX0602	2006	Jiangxi‐China	1,768	PCV2a	EF524541	Wang et al., 2009
DK1980PMWSfree	2007	Denmark	1,767	PCV2c	EU148503	Dupont et al., 2008
DK1987PMWSfree	2007	Denmark	1,767	PCV2c	EU148504	Dupont et al., 2008
MDJ	2007	Heilongjiang‐China	1,766	PCV2d	HM038031	Guo et al., 2010
LG	2008	China	1,768	PCV2a	HM038034	Guo et al., 2010
C6	2008	South Korea	702 ORF2 gene	PCV2b	EU450638	Franzo & Segalés, 2018
762,454	2008	England	702 ORF2 gene	PCV2b	KY806003	Grierson, Werling, Bidewell, & Williamson, 2018
P2425NT	2008	Vietnam	1,767	PCV2g	JX099786	Huynh et al., 2014
549‐QNa	2009	Vietnam	1,767	PCV2h	KM042398	Franzo & Segalés, 2018
TY1	2010	Taiwan‐China	1,768	PCV2a	HQ202949	Franzo & Segalés, 2018
PM163	2010	Brazil	1,767	PCV2c	KJ094599	Franzo et al., 2015
ZrBd_wb UKR	2010	Ukraine	1,767	PCV2g	KP420197	Franzo & Segalés, 2018
201207JS	2011	Jiangsu‐China	1,767	PCV2d	KX960929	Franzo & Segalés, 2018
BG0‐1	2011	Vietnam	1,767	PCV2h	JQ181592	Huynh et al., 2014
3196‐LE	2012	Slovakia	702 ORF2 gene	PCV2b	KP768478	Sliz, Vlasakova, Jackova, & Vilcek, 2015
PCV‐Y22	2012	China	1,767	PCV2d	KC515014	Franzo & Segalés, 2018
MZ‐9	2012	India	1,767	PCV2f	LC008135	Franzo & Segalés, 2018
CBI090	2013	Thailand	705 ORF2 gene	PCV2d	MF314285	Thangthamniyom et al., 2017
MZ‐5	2013	India	1,767	PCV2f	LC004750	Franzo & Segalés, 2018
AS‐2	2013	India	1,767	PCV2f	LC008137	Franzo & Segalés, 2018
USA/NE‐002/2015	2015	USA	1,777	PCV2e	KT870147	Franzo & Segalés, 2018
USA/43520/2015	2015	USA	1,777	PCV2e	KT795289	Harmon et al., 2015
KU‐1609	2016	South Korea	1,768	PCV2a	KX828215	Franzo & Segalés, 2018

### 2.2 Phylogenetic analyses of PCV2sequences

To provide better insight into the extent of genetic heterogeneity among PCV2 strains in southern China, a phylogenetic tree of the ORF2 gene was constructed with MEGA v.10.0.5 software using the neighbour‐joining (NJ) method (Kumar, Stecher, & Tamura, 2016). A bootstrap value was calculated using 1,000 replicates. The newly proposed method for genotyping by Franzo and Segalés (2018) was applied.

### 2.3 Analysis of antigenic structure of PCV2 ORF2 gene

It is widely accepted that the variation in the antigenic structure of the deduced capsid protein encoded by the ORF2 gene is important evidence for viral adaptability. Therefore, we performed an epitope cluster analysis via the newly developed cluster‐breaking algorithm with an identity threshold of 80% on ORF2 genes used in this study (http://tools.iedb.org/cluster2/). In addition, predictions of B‐cell epitopes, secondary structures and surface locations were also performed on this target gene by using the BepiPred method (Minin, Bloomquist, & Suchard, 2008). These synthetic datasets displayed the variation in this gene sequence in different PCV2 strains.

### 2.4 Nucleotide and amino acid substitutions analysis of PCV2 ORF2 sequences

Substitution rates of the nucleotide sequences and the deduced amino acid sequences of ORF2 gene products were analysed by the Tamura–Nei method using MEGA v.10.0.5. Tajima's neutrality test was performed to calculate the Θ, π and D values, which are the common indicators within the neutrality test that are used for evaluating the selection pressure on the group being tested, following the procedures described elsewhere (Nielsen, 2001). In addition, we gathered the frequencies of the amino acids used by PCV2 strains and the average frequencies and their standard deviations (*SD*) of each amino acid among different PCV2 genome sequences.

### 2.5 Recombination analysis of PCV2 sequences

To investigate the recombination rates, putative breakpoints and potential parental sequences of the PCV2 genomes, Recombination Detection Program (RDP v.4.97) was utilized according to the recommendations of previous studies (Mu et al., 2012). To further comprehensively confirm the identified recombinant events, the seven recombination detection methods, namely RDP, MaxChi, GeneConv, BootScan, SiScan, 3Seq and Chimaera, which are abbreviated to R, M, G, B, S, T and C, respectively, that had been implemented in the RDP4 software were employed again to ensure an acceptably low rate of false positives. The correlation parameters were identical to those reported by Li, He, et al. (2018)

## 3 RESULTS

### 3.1 Nucleotide sequences analysis

The sequence analysis showed that the complete genomes of these 92 PCV2 Chinese strains were 1,767 or 1,768 bp in length, while the ORF2 nucleotide sequences were 702 or 705 bp. The pairwise comparison analysis revealed that the nucleotide homologies of the complete genomes among these 92 PCV2 Chinese strains ranged from 93.7% and 100% (data not shown), while the pairwise similarities within 92 Chinese strains and 8 representative strains ranged between 91.1% and 100% (Table 3).

**TABLE 3:** Homology comparison of the ORF2 nucleotide sequence of PCV2 strains

Isolates	Strains for nucleotide sequence comparison							
	PCV2a	PCV2b	PCV2c	PCV2d	PCV2e	PCV2f	PCV2g	PCV2h
	AY556474	AY847748	EU148503	KX960929	KT870147	LC008135	FJ998185	KM042398
EF493839	95.2	98.2	94.7	95.6	90.2	95.6	95.2	96.2
EF493838	95.3	98.1	94.8	95.9	90.2	95.7	94.8	96.4
EF524532	99.1	95.3	93.9	94.5	89.8	95.7	92.5	95.6
EF524533	99.0	95.2	93.9	94.4	89.9	95.6	93.1	95.5
MH465418	95.1	99.1	94.5	96.2	91.0	95.8	95.3	96.1
MH465433	95.2	98.8	94.7	96.2	91.0	95.8	95.5	96.2
EF675241	94.8	95.9	94.3	98.7	91.8	95.3	97.3	96.1
EF675240	95.2	98.8	94.7	96.1	91.0	95.8	95.5	96.1
EF675239	95.5	98.5	95.1	96.0	90.5	96.0	95.1	96.6
EF675238	94.9	98.2	95.3	95.7	91.0	95.5	97.3	96.2
EF675237	95.4	98.4	95.1	96.0	90.5	95.9	96.0	96.5
MH465458	94.7	95.7	94.3	98.7	91.6	95.2	97.1	96.1
MH465420	95.2	97.8	94.5	96.9	90.7	95.5	95.7	96.3
MH465419	95.0	98.0	94.5	95.7	90.3	95.4	94.4	96.1
MH465398	95.4	98.7	94.7	96.3	91.0	95.9	95.5	96.3
MH465402	95.2	98.7	94.8	96.1	91.0	95.9	94.9	96.1
MH465460	94.4	95.7	93.9	99.2	91.0	95.1	95.7	96.5
MH465459	94.7	96.0	94.2	99.5	91.4	95.3	96.7	96.8
MH465422	95.2	98.1	94.5	96.9	90.8	95.5	95.8	96.4
MH465421	95.2	98.1	94.5	96.9	90.8	95.6	95.9	96.4
MH465399	95.3	98.9	94.7	96.1	90.9	95.8	95.6	96.1
KJ680370	94.6	95.7	94.1	99.4	91.4	95.2	96.6	96.6
KJ680369	94.7	96.0	94.3	99.6	91.5	95.4	96.8	96.8
KJ680368	94.7	96.0	94.3	99.6	91.5	95.4	96.8	96.8
KJ680367	94.7	95.9	94.2	99.5	91.4	95.3	96.8	96.8
MH465424	95.5	99.0	94.9	96.4	91.0	96.1	95.8	96.4
MH465423	95.3	98.8	94.7	96.2	90.9	95.9	94.8	96.2
MH756618	94.6	96.0	94.2	99.4	91.4	95.3	96.7	96.6
MH756617	94.6	96.4	94.2	98.8	91.4	95.3	95.5	96.5
MH465473	95.4	96.8	94.4	97.0	91.3	95.9	95.9	98.2
MH465461	94.7	95.9	94.3	99.5	91.5	95.3	96.6	96.8
MH465426	95.5	98.9	94.8	96.5	91.1	96.0	95.5	96.5
MH465425	95.5	98.8	94.8	96.5	91.1	96.0	95.6	96.5
MH756615	95.4	98.9	94.7	96.5	91.0	96.0	95.5	96.5
MH756613	94.7	96.1	94.2	99.7	91.5	95.5	96.7	96.9
MH756608	94.7	96.0	94.3	99.5	91.4	95.4	96.7	96.8
MH756610	94.7	96.0	94.3	99.6	91.5	95.4	96.8	96.8
MH756611	94.6	95.9	94.2	99.4	91.4	95.3	96.7	96.7
MH465475	94.7	96.0	94.3	99.5	91.3	95.3	96.7	96.8
MH465474	94.7	96.0	94.3	99.6	91.5	95.4	96.8	96.8
MH465437	95.6	98.9	94.9	96.6	91.1	96.1	95.7	96.6
MH465436	95.3	98.6	94.8	96.4	90.9	95.9	95.3	96.4
MH465435	95.3	98.7	94.5	96.1	90.8	95.7	94.6	96.1
MH465434	95.2	98.5	94.7	96.3	90.8	95.7	94.8	96.2
MH465490	99.0	95.1	93.9	94.6	89.9	95.6	96.4	95.6
KM245558	94.7	96.0	94.3	99.4	91.5	95.5	96.5	96.8
KJ956692	95.6	99.0	94.9	96.7	91.2	96.2	96.1	96.6
KJ956691	95.6	99.0	94.9	96.7	91.2	96.2	96.1	96.6
KJ956690	95.6	99.0	94.9	96.7	91.2	96.2	96.1	96.6
KJ956689	95.6	98.9	95.1	96.6	91.1	96.1	96.2	96.7
MH465464	94.7	96.0	94.4	99.4	91.4	95.3	96.5	96.8
MH465463	94.6	95.9	94.2	99.5	91.3	95.3	96.6	96.7
MH465462	94.6	95.9	94.2	99.4	91.4	95.3	96.6	96.7
MH465427	95.6	99.1	94.8	96.4	91.1	96.0	95.7	96.4
KY305198	94.6	96.6	94.3	99.3	91.7	95.5	96.6	96.5
MH756616	94.8	96.7	94.4	98.9	91.5	95.5	95.6	96.7
MH756614	94.8	96.3	94.4	99.4	91.2	95.4	95.9	96.9
MH465480	94.8	96.1	94.3	99.3	91.5	95.6	96.6	96.8
MH465479	94.6	95.9	94.2	99.4	91.3	95.3	96.6	96.6
MH465478	94.8	96.1	94.4	99.5	91.5	95.5	96.6	96.9
MH465477	94.7	96.1	94.3	99.5	91.5	95.4	96.7	96.8
MH756607	94.4	95.6	94.2	99.2	91.2	95.1	96.7	96.4
MH465476	94.8	96.8	94.4	99.0	91.6	95.6	95.7	96.7
MH465438	95.5	98.7	94.7	96.2	90.8	96.1	95.6	96.2
MH465403	95.5	98.8	94.8	96.5	91.1	96.0	95.5	96.5
MH465488	99.4	95.6	94.2	94.9	90.0	96.0	92.9	96.0
MH465487	97.8	95.0	93.8	94.6	90.2	96.4	92.2	95.5
MH465491	97.8	95.2	93.9	94.8	90.4	96.5	92.2	95.6
MH465467	94.6	95.8	94.2	99.4	91.2	95.2	96.6	96.6
MH465466	94.6	95.9	94.2	99.4	91.2	95.3	96.5	96.7
MH465465	94.6	95.8	94.1	99.4	91.6	95.3	96.6	96.6
MH465428	95.4	98.9	94.9	96.5	91.0	96.0	95.6	96.5
KY305202	95.2	98.8	94.7	96.1	90.8	95.7	94.8	96.2
KY305201	95.5	98.8	94.8	96.6	91.1	96.0	96.0	96.5
KY305200	94.5	95.9	94.2	99.3	91.3	95.2	96.3	96.6
KY305199	94.6	95.9	94.3	99.3	91.4	95.2	96.5	96.6
MH465481	94.3	96.1	93.9	98.2	91.2	94.9	94.6	96.2
MH756609	94.9	96.2	94.5	98.9	91.7	95.5	96.5	96.5
MH465443	94.8	95.9	94.3	99.5	91.4	95.3	96.7	96.8
MH465442	94.5	95.7	94.1	99.2	91.2	95.1	96.3	96.5
MH465471	94.4	95.7	93.8	98.9	91.0	95.1	96.2	96.4
MH465470	94.9	96.1	94.5	99.4	91.4	95.6	96.8	96.8
MH465469	94.8	96.6	94.3	98.8	92.0	95.5	95.5	96.6
MH465468	94.6	96.3	94.2	98.6	91.5	95.2	95.2	96.4
MH465431	95.1	98.3	94.5	96.3	91.2	95.6	95.0	96.2
MH465430	95.1	98.4	94.5	96.1	90.9	95.6	95.0	96.0
MH465429	95.4	98.9	94.7	96.3	91.2	95.9	95.6	96.4
KY305204	94.7	96.0	94.3	99.4	91.4	95.4	96.5	96.8
KY305203	94.9	96.1	94.3	99.5	91.5	95.5	96.6	96.9
MH465482	94.8	96.1	94.2	99.2	91.3	95.6	96.5	96.8
MH465472	95.2	96.5	94.8	99.3	91.9	95.9	97.0	96.9
MH465489	96.5	94.5	93.1	94.1	89.9	95.1	91.7	95.1

### 3.2 Phylogenetic relationships of PCV2 isolated in southern China

**FIGURE 2:**
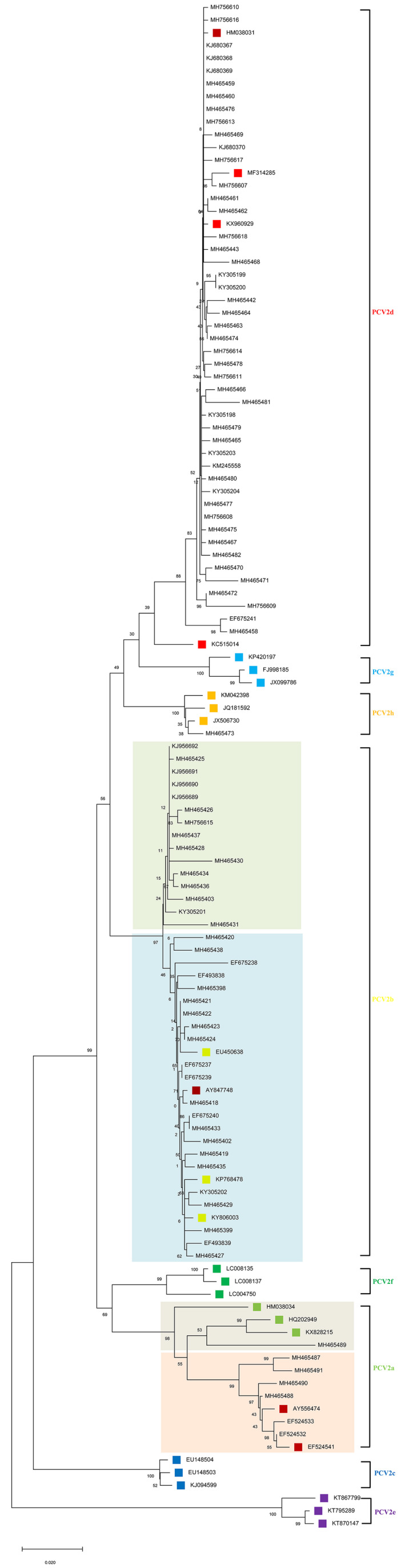
The phylogenetic tree was constructed by the neighbour‐joining method for 120 ORF2 sequences of PCV2 using MEGA v.10.0.5 software. Bootstrap values were calculated on 1,000 replicates. The 28 reference sequences are labelled by filled square

Based on the subgroup terminology in a previous report [13], the phylogenetic analysis indicated that 92 strains could be divided into four genotypes: PCV2a (7/92), PCV2b (37/92), PCV2d (47/92) and PCV2h (1/92), with PCV2d being the currently prevalent genotype (Figure 2; Table 1) [18]. Further research on the genotyping of these PCV2 strains found that PCV2 strains coming from the same or different regions may fall into the different or the same subgroups (e.g. MH465458 and MH465420; EF675240 and MH465419) (Table 1; Figure 1) (Wang et al., 2009; Zhang et al., 2014), and this discovery is similar to findings of a previous study in which the hypothesis was put forward that the prevalence of different PCV2 subgroups does not have a significant geographical characteristic (Li et al., 2010). Consistently, the genetic distances between the Chinese PCV2 strains were relatively far away from one another (data not shown) and the strains of each genotype were interspersed with the same genotypic PCV2 strains worldwide.

### 3.3 Amino acid analysis

**FIGURE 3:**
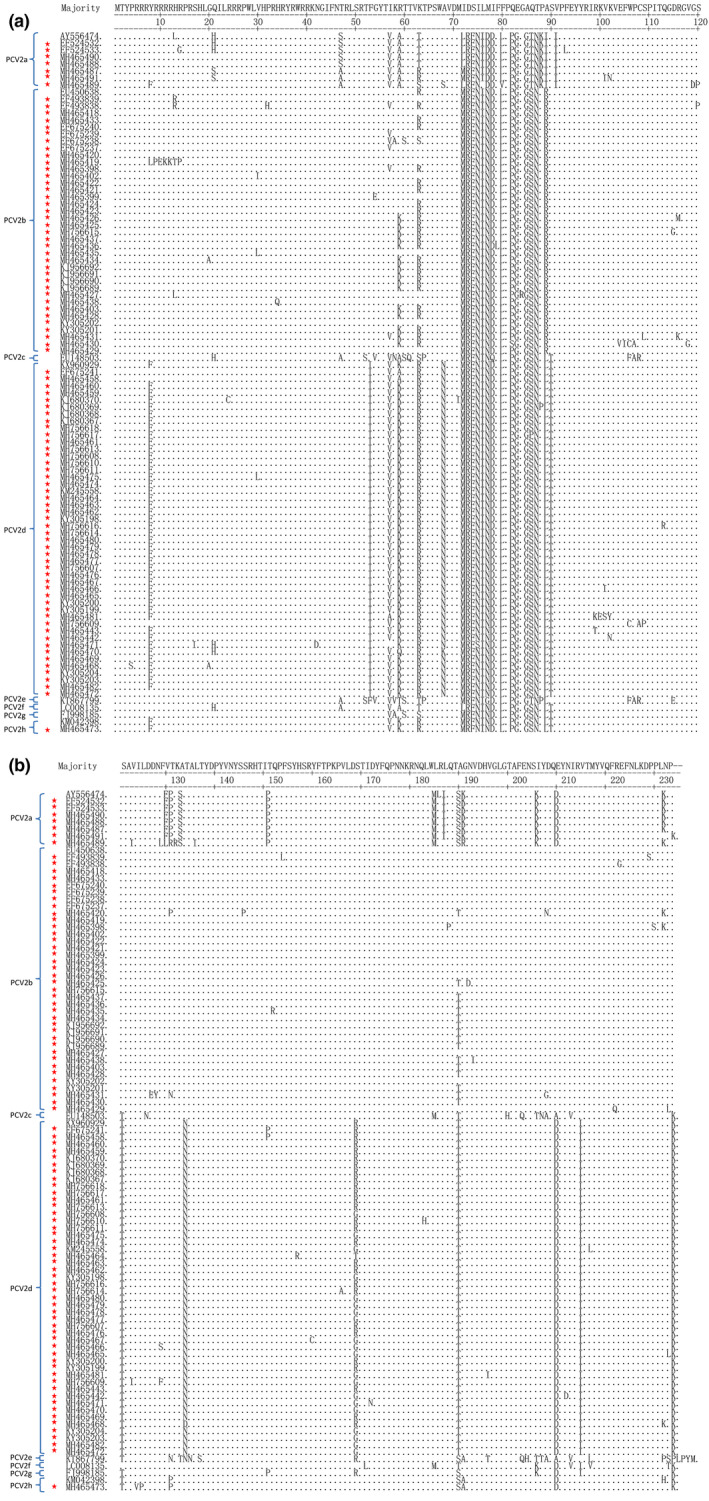
Pairwise alignment of amino acid sequences for the capsid protein of 100 PCV2 strains including 92 Guangxi strains as well as 8 selected representative sequences in the study. Residues that are exactly consistent with the consensus are indicated with dots. BJW is used as the majority sequence for this alignment (AY847748). The 92 Guangxi Beibu Gulf strains are marked with pentagram

**FIGURE 4:**
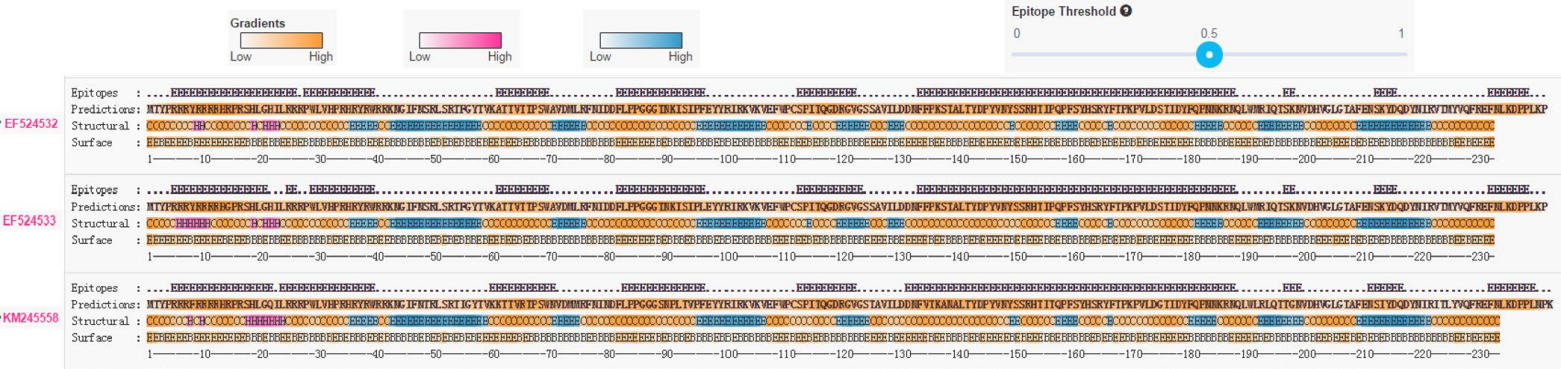
Epitopes and predictions in genes of part of the species. Epitopes: Positions above epitope threshold. Predictions: The protein sequence is displayed with an orange gradient, illustrating BepiPred‐2.0 predictions. Structural: Helix (H ‐ pink probability gradient), Sheet (E ‐ blue probability gradient) and Coil (C‐ Orange probability gradient) predicted. Surface: Buried(B)/Exposed(E) and orange gradient illustrating predicted relative surface accessibility. The 3 representative Guangxi Beibu Gulf strains are shown here

To investigate variation in the amino acid sequences of the putative capsid protein, the deduced amino acid sequences of the ORF2 gene of 100 PCV2 strains, including the 92 Chinese strains and 8 selected representative strains, were subjected to pairwise alignment. As shown in Figure 3, the divergence at the amino acid level was greater than that of the nucleotide sequence (Table 3), displaying lower similarity that ranged from 78.6% to 100% (data not shown). Analysis of amino acid substitutions in ORF2 genes indicated that there were several major regions, located at positions 52–68 and 185–191 within the Chinese PCV2 ORF2 sequences, of high heterogeneity in this study, in spite of ubiquitous variations in the whole capsid protein sequence. In addition, the ORF2 amino acid variations have also been found at several other positions, such as at 8 (tyrosine to phenylalanine or leucine), 13 (histidine to leucine, arginine or threonine), 57 (isoleucine to valine), and 63 (lysine to threonine, serine or arginine). The nucleotide replacement rate analysis showed that the amino acids with greatly changed operating frequencies during the evolutionary process of PCV2 were asparagine, serine and lysine, suggesting that these amino acids may be involved in PCV2 evolution, although many important questions remain. The results of Tajima's neutrality test demonstrated that the indicator *D* value was significantly lower than zero, implying that the group size of PCV2 Chinese strains was potentially enlarging or these strains were possibly suffering some kind of directional selection. Epitope cluster analysis with the BepiPred method disclosed that there may be also several other new short epitopes, located at positions 5–40, 60–70, 80–98, 113–123, 210–213 and 220–227, which distributed dispersively across the whole capsidprotein sequence (Figure 4). This information may provide new evidence for PCV2 phenotyping.

### 3.4 Recombination analysis

**FIGURE 5:**
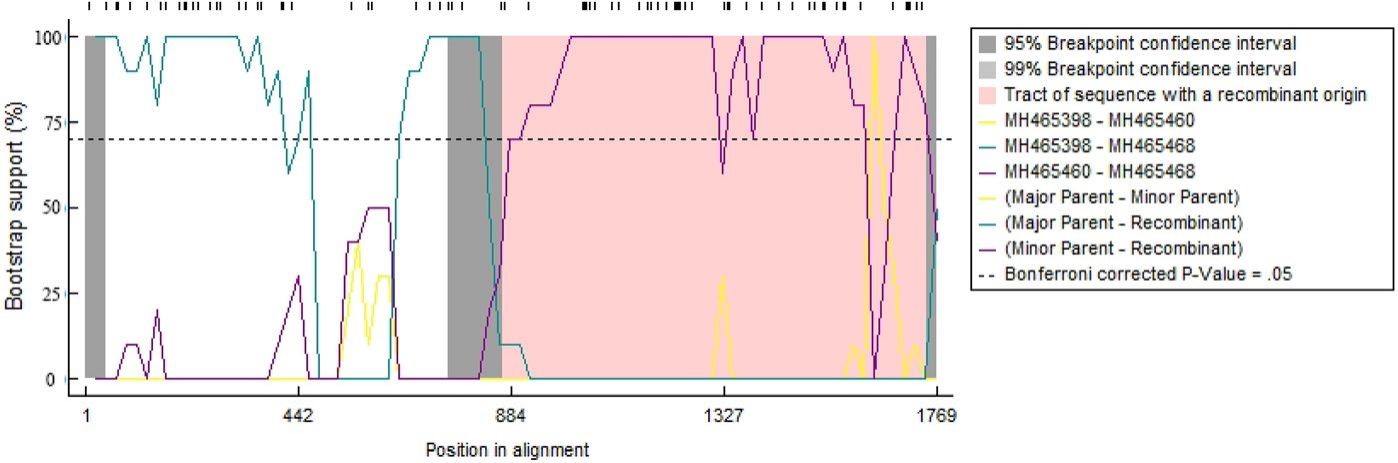
Identification of the recombination events. The most representative recombinant (MH465468) derived from recombination between MH465398 and MH465460 was shown by the means of the BootScan method with a window size 30. The *y*‐axis refers to bootstrap support, *x*‐axis represents the positions of informative sites, and the left and right bounds of the pink tract region indicate breakpoint positions

**FIGURE 6:**
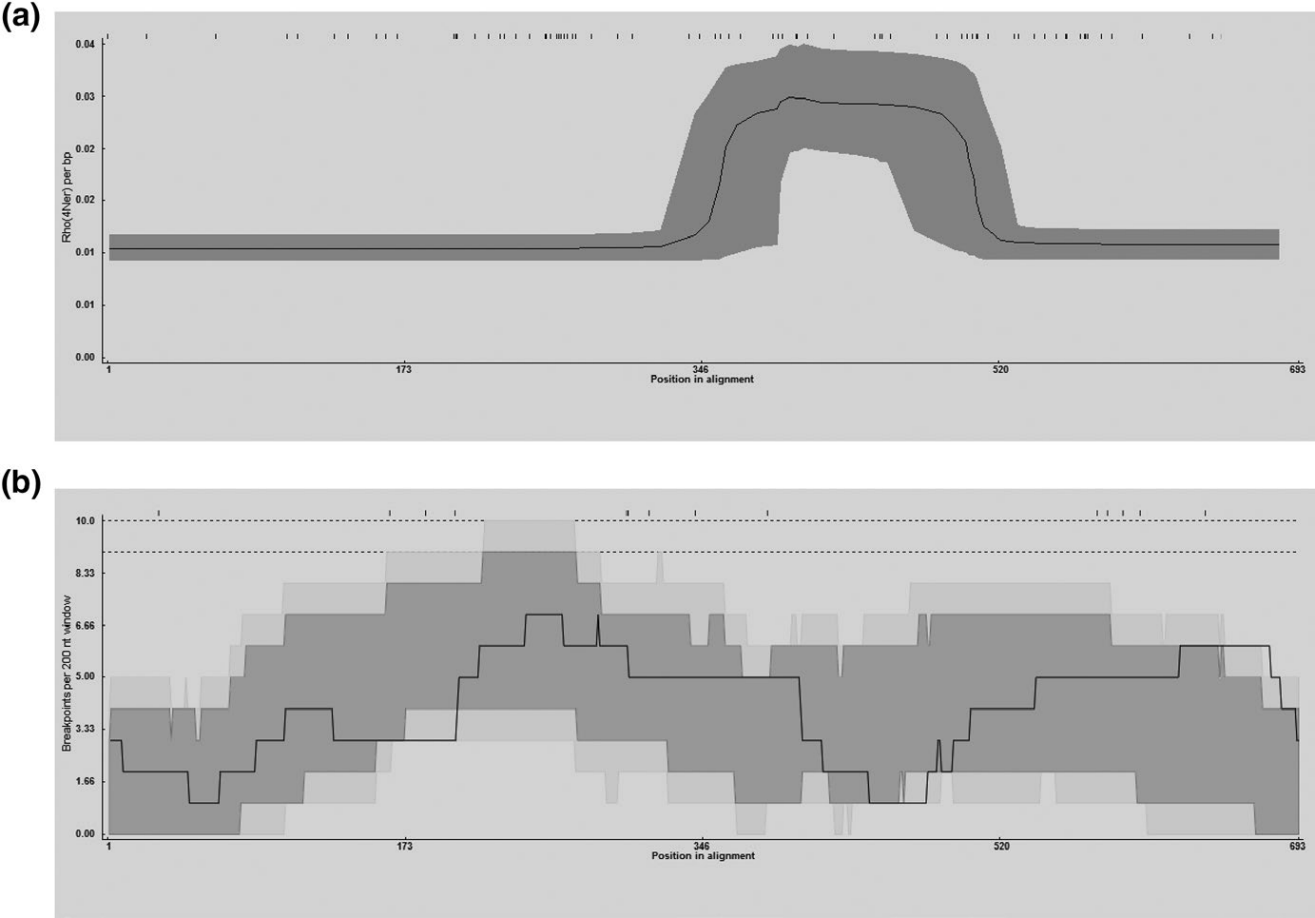
Recombination rates and distribution of breakpoints. The x‐axis refers to the positions of informative sites. The *y*‐axis in A represents Rho (4Ner) per base pair and the *y*‐axis in B represents breakpoints per 200‐nucleotide window

Based on the analysis performed using the RDP v.4.97 software, a total of 18 potential recombination events were detected within all 92 of the analysed PCV2 strains from southern China, and only the five recombination events that had been confirmed by at least three of the seven methods with *p* value cut‐off of 0.05 were reported here (Table 4). The only recombination event that was confirmed separately using the seven methods implemented in RDP4 was shown in Figure 5. Three of the recombination events likely involved direct exchanges of genetic material contained in the PCV2b and PCV2d sequences, although no evidence of the exchange of PCV2b and PCV2a sequences was detected. It is quite clear that the PCV2h sequences (MH465473) contained evidence of having acquired sequences from PCV2b sequences (EF675238 and MH465430). All the five reported recombination events involved transfers of large fragments of the ORF2 gene, a different group of the five recombination events involved transfers of small fragments of the ORF1 gene (see Figure 5 as an example). Specifically, it was observed that the recombination events primarily targeted the region between nucleotides 1,215–1,389 within the ORF2 gene (Figure 6).

**TABLE 4:** Results of the identification of recombination events among 92 PCV2 genomes isolated from the southern China using the RDP, MaxChi, GeneConv, BootScan, SiScan, 3Seq and Chimaera algorithms, respectively

Event number	Recombinant/genotype	Major parent/genotype	Minor parent/genotype	Detection methods						
				R	G	B	M	C	S	T
1	MH465421/PCV2b	MH465460/PCV2d	MH465423/PCV2b	−	+	+	+	+	+	+
2	MH465420/PCV2b	MH465458/PCV2d	MH465418/PCV2b	+	−	−	+	−	+	+
3	MH465468/PCV2d	MH465398/PCV2b	MH465460/PCV2d	+	+	+	+	+	+	+
4	EF493839/PCV2b	MH465473/PCV2h	MH465399/PCV2b	−	−	+	+	+	+	+
5	MH465473/PCV2h	EF675238/PCV2b	MH465430/PCV2b	−	−	−	+	−	+	+

## 4 DISCUSSION

Homogeneous analysis demonstrated that the overall diversity of PCV2 strains in southern China is still low, as the lowest sequence similarity observed between any two Chinese strains was 93.7%, which is similar to the sequence similarity of 94.6% found in a previous study in 2009 (Harmon et al., 2015) and the sequence similarity of 92.7% described in another report in 2012 (Table 3) (Mu et al., 2012). However, phylogenetic and other genetic variation analyses that have aimed to elucidate the evolution and spread of PCV2 are still compelling, because the ORF2 gene of PCV2 is relatively free of recombination, which is a prominent feature of PCV2 evolution (Olvera et al., 2007).

Based on the ORF2 gene, a phylogenetic tree was generated using the neighbour‐joining method. The results indicated that the 92 PCV2 isolates from the southern China could be grouped based on two major genotypes (PCV2b and PCV2d), with PCV2d being the more predominant PCV2 genotype in this region (Figure 2 and Table 1). Meanwhile, we found that the ORF1 region in three Chinese strains was sharing high homology with that of a strain of PCV2c that was first recognized in Denmark and reported in a recent study, although no evidence of the presence of PCV2c was found in this region (Liu, Wang, Zhu, Sun, & Wu, 2016). Currently, however, whether the three Chinese strains derived from Denmark by international trade transportation or genovariation are still unclear. Notably, the size of the PCV2d group is significantly larger than those reported by Wang et al. (2009) and Mu et al. (2012), and is significantly larger than the PCV2b group, which indicates that PCV2d is undergoing an increase in population size due to some directional selection and is becoming the predominant genotype in China. This conclusion was further confirmed by a nucleotide replacement rate analysis that indicated that the *D* value derived from Tajima's neutrality test was significantly less than zero. Nevertheless, all of the 92 PCV2 strains analysed in this study were collected after 2006 (Table 1) (Wang et al., 2009; Zhang et al., 2014), which coincided with an increase in the severity of PCVD cases in China (Li et al., 2010). Moreover the findings are consistent with a previous report that indicated that the genotypic shift from PCV2b to PCV2d likely occurred in approximately 2010 (Franzo & Segalés, 2018; Yang et al., 2018). Coincidentally, global genetic analysis indicated that the PCV2 evolution trace was PCV2a to PCV2b to PCV2d and has occurred in many countries (Franzo & Segalés, 2018). In the USA, a variant PCV2 mutant strain designated as mPCV2b, and now grouped in PCV2d, was detected in several PCVD cases in 2012 and the prevalence rate of it appears to have increased in recent years, indicating that there is an ongoing genotype shift occurring from PCV2b to PCV2d on a global scale in these subsequent years (Franzo & Segalés, 2018; Jiang et al., 2017; Xiao, Halbur, & Opriessnig, 2012). Currently, there are series commercial vaccines that are PCV‐2b or PCV‐2a based are extensively used in this region, which phenomenon may also contribute to the evolution of PCV2 from PCV2b to PCV2d genetic subtype, as PCV2 virus is able to evolve by way of mutation and recombination in response to the wide‐spread application of these vaccines. Further phylogenetic analysis indicated that the PCV2b strains obtained from different parts of the region were closely related to each other but were more distantly related to other Chinese and foreign PCV2b strains. This finding suggests that these distinct PCV2b genotypic strains originated in and are endemic to the southern China. However, it is worth pondering whether there are more genotypes should be proposed as two different clusters that are marked by shadows with pale yellow and wathet blue, respectively are found within PCV2b genotype, and another two different clusters that are marked by shadows with grey and pink separately were also discovered within PCV2a genotype (Figure 2).

As reported previously, the Cap protein is considered the most variable structural protein of PCV2, and the amino acid variation in this region might be associated with pathogenicity and/or immunogenicity (Fenaux, Opriessnig, Halbur, Elvinger, & Meng, 2004; Mu et al., 2012). Therefore, an amino acid alignment of the Cap protein, which is encoded by the ORF2 gene, was also conducted. Our results show that there are two major regions of variation, residues 52–68 and 185–191, that correspond to two of the three dominant immunoreactive areas identified by Lekcharoensuk et al. (2004). In addition, these data show that patterns specific to each group exist, such as Chinese PCV2 strains clustered within PCV2h had one amino acid marker region located at positions 57–63 and two specific amino acid variations found at positions 124–125. However, it is strange that less variation or no significant differences were observed within the newly reported antigenic recognition regions (residues 117–131, 132–146, 156–162, 195–202 and 230–233) (Li et al., 2010; Shang et al., 2009). All of the ORF2 genes of the 47 PCV2d strains from the southern China encoded 234 aa, while the vast majority of the remaining strains encoded 233 aa, which is the same number that was encoded by ORF2 in the PCV2 strains isolated from other parts of the world (Figure 2). The extra amino acid encoded by Chinese PCV2d strains is lysine and is the penultimate residue of the Cap protein; this extra residue was identical in other PCV2d strains identified by Wang et al. (2009). Further observation indicated that some amino acid mutations of the Cap protein could be used for PCV2 genotyping, especially for differentiating the novel genotype PCV2d from other genotypes, as many amino acid variations found at specific positions were unique to the PCV2d genotype. For example, the ORF2 amino acid variations at positions F53I and A68N were unique to the PCV2d genotype. Thus, whether the above‐mentioned amino acid variations at specific positions contribute to the pathogenicity and virulence of PCV2 strains requires further investigation. In addition, some previous reports have demonstrated that there is a highly conserved putative N‐glycosylation site within the PCV2 Cap protein at amino acid residues 143–145 (NYS), which may have a tight link with the host immune responses due to its potential impact on the immunogenicity of Cap‐based DNA vaccines (Gu et al., 2012). A similar phenomenon occurred in the antigenic epitope 26‐RPWLVHPRHRY‐36 in the nuclear localization signal region of the PCV2 Cap protein (Guo, Lu, Huang, Wei, & Liu, 2011). Interestingly, the N‐terminus of the Cap protein encompassing the nuclear localization signals in all the 92 PCV2 sequences was also found to be fairly well conserved (Figure 3), further confirming the inferred importance of this site. Epitope cluster analysis revealed that there were eight potential epitopes within the Cap protein in the 92 PCV2 strains (Figure 4), of which two epitopes (residues 5–40 and 130–190) were mainly related to antigen recognition and were highly conserved in all of the 92 analysed PCV2 sequences. We noted, however, that the remaining short epitopes do not correspond to the six dominant immunoreactive areas identified in other studies, and this finding may therefore present new evidence for PCV2 adaptability.

Genetic recombination is an important evolutionary mechanism that generates diversity in PCV2. For instance, a novel PCV genotype that had apparently arisen through recombination between the ORF2 genes in the PCV2a and PCV2b strains was discovered by Cai et al. (2012). In this study, we have identified five recombinant events that occurred primarily within the ORF2 and/or ORF1 gene; four out of the five recombination events resulted in inter‐genotype recombination, whilst only one recombination event resulted in intra‐genotype recombination between PCV2b sequences (Table 4), which implies that recombination has likely played an important role in the genetic diversity of PCV2 in the southern China. Coincidentally, the finding was consistent with the previously determined result that PCV2 strains have been involved in both inter‐ and intra‐genotype recombination events (Mu et al., 2012). Due to the obvious evidence of recombination between Chinese PCV2a and PCV2b genotypes detected in other studies, we propose that recombination between different PCV2 strains in China should not be restricted to PCV2b lineages and that recombination that occurred in the ORF2 genes between different PCV2 genotypes has likely contributed to the difference in viral pathogenicity. In addition, the southern China may have acquired PCV2h strain from Vietnam by international trade, as three PCV2h representative strains were all found in Vietnam and the China borders Vietnam on the southwest; however, the specific details regarding how this occurred are still unclear, but at least further demonstrating clearly that the recombination patterns between different PCV2 strains in China are complicated and more careful research is needed in the future.

## 5 ETHICS STATEMENT

All the 120 PCV2 full‐genome sequences were retrieved from GenBank and thereby no ethical approval was required.

## CONFLICT OF INTEREST

The authors declare that they have no conflict of interest.

## AUTHOR CONTRIBUTION

**Qi‐zhuang Lv:** Conceptualization; Funding acquisition; Writing‐original draft. **Tao Wang:** Data curation; Methodology. **Jia‐hua Deng:** Data curation; Methodology. **Yan Chen:** Formal analysis; Software. **Qiu Yan:** Formal analysis; Software. **Dao‐bo Wang:** Conceptualization; Funding acquisition; Supervision; Validation; Writing‐review & editing. **Yu‐lin Zhu:** Conceptualization; Supervision; Validation; Writing‐review & editing.
